# Flow-synchronized nasal intermittent positive pressure ventilation for prevention of extubation failure in neonates: a review of literature and a case series of neonates with congenital diaphragmatic hernia successfully managed with this strategy

**DOI:** 10.1186/s13052-025-02143-z

**Published:** 2025-12-02

**Authors:** Sara Ronci, Stefano Caoci, Camilla Gizzi, Flaminia Calzolari, Irma Capolupo, Domenico Umberto De Rose, Chiara Maddaloni, Ludovica Martini, Ferdinando Savignoni, Corrado Moretti, Andrea Dotta

**Affiliations:** 1 Neonatal Intensive Care Unit, “Bambino Gesù” Children’s Hospital IRCCS, Piazza S. Onofrio, 4, Rome, Italy; 2 Department of Neonatology and NICU, Ospedale Sant’Eugenio, Rome, Italy; 3 Union of European Neonatal and Perinatal Societies (UENPS), Milan, 20143 Italy; 4 Department of Pediatrics, Policlinico Umberto I, Sapienza University, Rome, Italy

**Keywords:** sNIPPV, nIPPV, Extubation failure, CDH, Respiratory support, Newborn

## Abstract

**Background:**

Advances in neonatal medicine have improved survival rates in neonatal intensive care units, especially for high-complexity cases like congenital diaphragmatic hernia (CDH). Despite these advances, managing respiratory failure in CDH infants remains challenging due to lung hypoplasia, respiratory insufficiency, and pulmonary hypertension. Lung-protective ventilation strategies are crucial to minimize ventilator-induced lung injury, but weaning them from invasive mechanical ventilation remains complex, and extubation failure rates are high.

**Case presentation:**

This retrospective case series describes the use of Flow-Synchronized Nasal Intermittent Positive Pressure Ventilation (sNIPPV) in four neonates with surgically corrected left CDH admitted to the “Bambino Gesù” Children’s Hospital in Rome (Italy) from 2022 onwards. Flow-sNIPPV was administered using the Giulia^®^ ventilator, which features a flow sensor for synchronization. We observed improved outcomes in terms of extubation success, in comparison to non-synchronized Nasal Intermittent Positive Pressure Ventilation (NIPPV). Synchronization reduced work of breathing (WOB), improved lung ventilation, and enhanced gas exchange without increasing ventilation-related complications. Additionally, this study reviews the current literature on the use of sNIPPV in neonates, highlighting the need for more research on its role in weaning post-extubation in CDH infants.

**Conclusions:**

Flow-sNIPPV shows promise in preventing extubation failure in neonates with CDH by improving ventilation and reducing WOB. Synchronization enhances lung ventilation, stabilizes the chest wall, and may reduce thoraco-abdominal asynchrony in CDH infants. While the findings are promising, larger multicenter studies are required to confirm the efficacy and safety of sNIPPV as a routine weaning strategy in CDH neonates after repair surgery.

## Introduction

Along with continuous advances in neonatal medicine, survival rates in intensive care units (NICUs) have significantly improved [1]. This has resulted in an increasing need to care for high-complexity infants, including newborns with congenital diaphragmatic hernia (CDH).

Respiratory failure in neonates is a common clinical condition associated with a high risk of morbidity and mortality [2].

The international guidelines recommend non-invasive ventilation (NIV) as a first-line respiratory support modality for its superiority in preventing death or bronchopulmonary dysplasia (BPD) compared to invasive mechanical ventilation [3]. In recent years, lung-protective ventilation has been implemented, focusing on avoiding ventilator-induced lung injury (VILI), mainly using volume-target ventilation [4]. In CDH infants, the difficult management is due to a combination of lung hypoplasia, respiratory insufficiency, pulmonary hypertension (PH), and hemodynamic instability [5].

Although a lung-protective strategy can mitigate lung damage, it can never completely prevent it. It is for this reason that newborns should be weaned from invasive support as soon as possible. In the literature, there is a lack of studies about the best weaning approach from invasive support; therefore, an important scientific effort is underway to determine the best post-extubation ventilatory strategy.

Several works have proposed a role for Neurally Adjusted Ventilator Assist (NAVA) and Non-Invasive Neurally Adjusted Ventilator Assist (NIV-NAVA) in patients with CDH [6], but to date, the presence of pleural effusion, common elements in the postoperative period, seems to represent an obstacle for this modality. Among the various NIV strategies, nasal intermittent positive pressure ventilation (NIPPV) is known to allow the transmission of higher mean airway pressure (MAP) [7]; the possibility of synchronizing this modality with the spontaneous breathing acts of the newborn could be an additional weapon [8, 9].

In our NICU of Bambino Gesù Children’s Hospital IRCCS in Rome (Italy), we care for many surgical newborns and are a referral center for managing CDH. Recently, we have observed a lower extubation failure rate after adopting the Giulia^®^ ventilator (Ginevri srl Medical Technologies, Rome, Italy). This device enables synchronized nasal positive pressure ventilation (sNIPPV) mode.

In this paper, we described 4 cases of neonates affected by CDH in whom sNIPPV effectively prevented extubation failure. Moreover, we reviewed the current scientific literature regarding the use of sNIPPV in neonates.

## Methods

### Case series

This is a retrospective case series of four neonates with left CDH hospitalized in our NICU from 2022 onwards and weaned from mechanical ventilation with NIPPV and sNIPPV. We compared the efficacy of the two non-invasive ventilation strategies.

NIPPV was administered using a Leoni^®^ ventilator (Löewnstein Medical), whereas sNIPPV was administered adopting the Giulia^®^ ventilator (Ginevri srl Medical Technologies, Rome, Italy).

Information on patients’ characteristics and data about blood gas values, vital signs, ventilatory parameters, and lung ultrasonography (LUS) patterns (based on the specific CDH-LUS score that we previously described [10]) and chest X-ray were systematically collected during both the NIV modalities. In particular, lung ultrasonography was performed using a modified six-field subdivision rather than the classic Brat method. This approach was chosen to allow a more detailed evaluation of regional lung aeration, particularly in neonates with congenital diaphragmatic hernia, who often present heterogeneous lung involvement. The six-field score provides improved sensitivity for detecting localized areas of atelectasis or lung re-expansion during the post-extubation period.

Parents signed a written informed consent form regarding publishing data about their infants.

### Review of literature

We reviewed available studies on using sNIPPV in neonates through an extensive search in the MEDLINE database (via PubMed), performed up to 30th November 2024. The keywords “sNIPPV” AND “neonate” OR “newborn” were searched as entry terms. Papers written in languages other than English were excluded.

## Results

### Case series

Demographic data of the four patients are reported in Table 1. The clinical features during the NIPPV and after during the sNIPPV are shown in Table 2. All patients survived.

**Table 1:** Demographic data

	P1	P2	P3	P4
Sex	M	M	M	M
GA	40	38	35	35
GA at diagnosis of CDH	20	21	20	NA
LHR O/E	44,1	45,4	40,6	NA
MSA	33	24	35	NA
Liver UP	no	no	no	yes
Weight at birth (g)	3500	3100	2235	3000
APGAR at 5 min	5	8	8	NA
HFOV	yes	yes	yes	yes
Pulmonary Hypertension	yes	no	no	yes
Pulmonar vasodilators	no	no	no	yes
Vasopressor support	no	yes	yes	yes
Inotropic support	yes	yes	yes	yes
Hours of life at surgery	96	48	96	72
Type of defect	Left, B	Left, C/D	Left, B	Right, C/D
Post-surgery thoracic effusion	yes	yes	yes	yes
Thoracic dreinage	no	no	no	yes
Day of life at successful extubation	6	6	6	18
Days on NIV	10	4	24	NA
Day of life at discharge	39	31	39	NA

*GA* gestational age, *CDH* congenital diaphragmatic hernia, *LHR O/E* lung-to-head ratio observed-to-expected, *MSA* mediastinal shift angle, *HFOV* high frequency oscillatory Ventilation, *NIV* Non invasive ventilation

**Table 2:** Clinical features of the four patients during NIPPV and sNIPPV

	NIPPV								sNIPPV							
	pHc	pCO2c	paO2c	IR	SR	NF	RR	LUS	pHc	PCO2c	paO2c	IR	SR	NF	RR	LUS
P 1	7,23	72	49	+	+	+	85	18	7,41	52	56	-	-/+	-	45	15
P 2	7,28	68	57	+	+	-	75	16	7,35	54	59	-	-	-	40	11
P 3	7,13	64	66	+	+	-	80	24	7,38	50	69	-	-	-	42	17
P 4	7,2	107	42	+	+	+	85	24	7,39	46	60	-	+	-	45	18

*IR* intercostal retraction, *SR* subdiaphragmatic retraction, *NF* nasal flaring, *RR* respiratory rate, *c* capillary, *LUS* lung ultrasound score, *NIPPV* nasal intermittent positive pressure ventilation, *sNIPPV* synchronized nasal intermittent positive pressure ventilation

#### Case 1

The first patient (P1) was born at 40 weeks of gestational age (GA) via cesarean section for prenatal diagnosis of left CDH with an observed-to-expected lung-to-head ratio (LHR O/E) of 49.1% and a mediastinal shift angle (MSA) of 33°.

The patient was intubated at birth and required High-Frequency Oscillatory Ventilation (HFOV) and therapy with milrinone for PH and right ventricular dysfunction for 72 h.

**Figs. 1:**
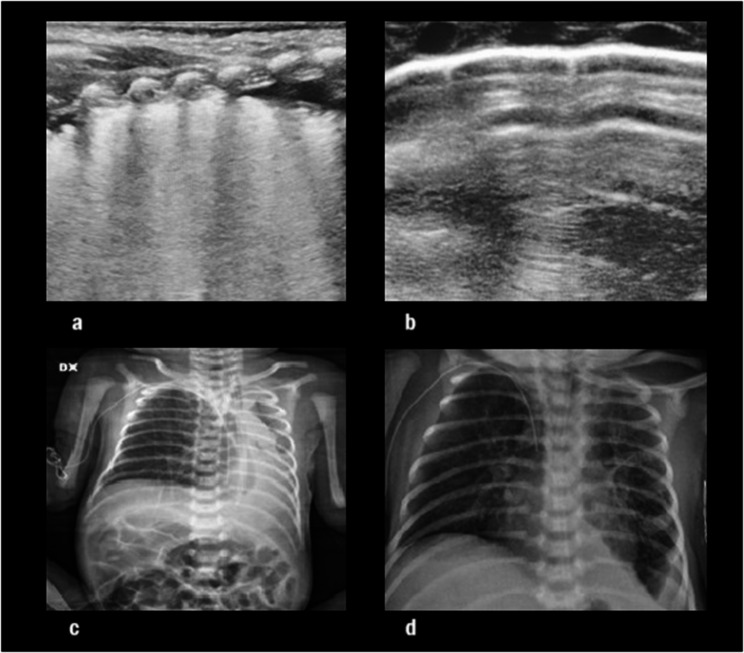
**a**, **b**, **c**, **d**. P1 LUS imaging during NIPPV (**a**) and sNIPPV (**b**); P3 chest X-ray during NIPPV (**c**) and sNIPPV (**d**)

Surgical correction was performed on the 4th day of life, revealing a type B/C defect. On the first post-operative day, the patient was treated with synchronized intermittent positive pressure ventilation (SIPPV). He developed a thoracic effusion that did not require drainage. On the 6th day of life, the patient was extubated and placed on NIPPV. However, respiratory acidosis, respiratory distress, and tachycardia were observed a few hours after extubation. The chest x-ray and the LUS showed poorly aerated lungs, with the left lung almost completely atelectatic (shown in Fig. 1a).

The ventilator parameters were progressively increased until reaching a peak inspiratory pressure (PIP) of 30 cmH2O, a positive end-expiratory pressure (PEEP) of 8 cmH2O, and a respiratory rate (RR) of 45.

After 10 h, to avoid reintubation, sNIPPV was attempted using the Giulia^®^ ventilator (initial parameters: PIP 26 cm H2O, PEEP 7 cm H2O, Back-Up Rate (BUR) 40, inspiratory time (IT) 0.40 s, and flow of 9 L/min). CO2 blood gas values decreased, and the patient’s respiratory dynamics improved. A LUS performed 12 h after initiating sNIPPV showed moderate lung aeration improvement (Fig. 1b).

The neonate was weaned from NIV after ten days. During the hospitalization, he underwent serial head ultrasound scans and Magnetic Resonance Imaging (MRI) of the brain at 37 days of life, and all were normal.

The baby was discharged on the 39th day of life without any respiratory support. 

#### Case 2

The second patient (P2) was a male born at 38 weeks of GA via elective cesarean section due to a prenatal diagnosis of CDH at 21 weeks of gestational age with an LHR O/E 67.4%, MSA of 24°, liver down.

The patient was intubated in the delivery room, and HFOV was initiated.

He initially required hemodynamic support with milrinone, vasopressin, and dopamine. Around 48 h of life, the baby showed a sudden clinical worsening with abdominal distension. An abdominal x-ray revealed pneumoperitoneum, and therefore, despite the incomplete clinical stability, surgery was performed. The laparotomy revealed an isolated intestinal perforation; repair of the diaphragmatic type B defect was also performed.

Seventy-two hours post-operation, the patient started his respiratory support with conventional ventilation with a progressive reduction of inotropic and vasopressor support until discontinuation. He was extubated on the 6th day of life and moved to NIPPV. A few hours after extubation, he began to exhibit respiratory distress with an increase in blood CO2 levels. A LUS showed reduced lung ventilation, especially on the left side, and the chest x-ray confirmed poor lung aeration. Therefore, the patient was shifted to sNIPPV with the Giulia^®^ ventilator with initial parameters of PIP 26 cm H2O, PEEP 7 cm H2O, BUR 40, IT 0.40s, and a flow of 9 L/min. The LUS control improved lung aeration, as confirmed by the chest x-ray. Blood gas CO2 values returned to normal limits, and the newborn’s respiratory dynamics rapidly improved. It was possible to wean off NIV within 48 h completely. During the hospitalization, he underwent serial head ultrasound scans and brain MRI at 28 days of life, and all were normal. The baby was discharged on the 31st day without any respiratory support.

#### Case 3

The third patient (P3) was a male premature neonate born at 35 weeks of GA via elective cesarean section due to preterm premature rupture of membranes at 33 weeks of GA. Prenatal diagnosis of CDH was made at 20 weeks of GA, with an MSA of 35° and an LHR O/E of 31,8%, liver down. Corticosteroid prophylaxis was administered.

The baby was intubated at birth and supported for the first hours by HFOV and then switched to SIPPV. Surgical correction was performed on the 4th day of life, revealing a type B defect. The postoperative course was characterized by the formation of a left thoracic effusion, which did not require drainage.

At 48 h post-surgery, the first extubation attempt was made with a switch to NIPPV with initial ventilatory settings of PIP 25 mH2O, PEEP 6.5 cmH2O, RR 40, inspiratory time IT 0.40 s, flow 8.5 L/min. This attempt failed after a few hours due to complete atelectasis of the left lung (shown in Fig. 1c), respiratory distress, and respiratory acidosis. The patient also presented severe discomfort that required significant sedation.

The second extubation attempt was performed after 72 h with a switch to sNIPPV using the Giulia^®^ ventilator, with initial ventilatory settings of PIP 20 cmH2O, PEEP 6.5 cmH2O, BUR 35, flow 8.5 L/min, IT 0.4s. Respiratory dynamics remained stable, as did blood gas values. LUS performed 24 and 72 h after starting sNIPPV showed a progressive improvement in lung aeration and the chest X-ray (Fig. 1d).

Improved patient comfort enabled rapid weaning from analgesia.

The baby required a total of 24 days of NIV; a course of corticosteroids was administered orally, and he was finally discharged on the 41st day of life without any respiratory support.

#### Case 4

The fourth patient (P4) is a male born at 35 weeks of GA via vaginal delivery. Prenatal diagnosis of right CDH, no other prenatal data available. Due to severe respiratory insufficiency and severe PH not responsive to maximal pharmacological therapy, Veno-Arterial Extracorporeal Membrane Oxygenation (VA-ECMO) was initiated on the second day of life.

The diaphragmatic hernia repair surgery was performed on the third day of life, with the detection of a C/D defect, corrected by placing a cone-shaped Dacron patch. A thoracic drain was left in place. Weaning from VA-ECMO began on the 7th day of life, with VA-ECMO removal on the 9th day. After the defect repair and the cessation of extracorporeal circulation, it was possible to progressively reduce inotropic, vasopressor, and pulmonary vasodilator support until complete discontinuation on the 14th day of life.

The postoperative course was complicated by a chylous pleural effusion, which required multiple aspirations through the chest drain and treatment with octreotide. The chest drain was removed on the 20th day of life.

The newborn was supported with HFOV until the 15th day of life, then with conventional ventilation. On the 18th day of life, the first extubation attempt was made, transitioning to NIPPV. After 4 h, the newborn developed severe respiratory distress and hypercapnia. Despite the progressive increase in ventilatory parameters, the respiratory dynamics did not improve.

A LUS revealed complete right lung atelectasis. Therefore, he was re-intubated and placed back on SIPPV. On the 20th day of life, a second extubation attempt was made with sNIPPV using a Giulia^®^ ventilator with initial ventilatory parameters of PIP 25 cmH2O, PEEP 7 cmH2O, BUR 40, IT 0.40s. Following extubation, the newborn’s respiratory dynamics remained stable, blood gas values stayed within normal limits, and LUS showed progressive improvement in the lung’s aeration. Moreover, reducing and discontinuing analgosedation was possible due to improved comfort. The infant is still hospitalized in our unit and is currently being weaned off NIV.

### Review of the literature

Thirty-two articles were initially retrieved, and then full texts of records deemed eligible for inclusion were assessed. References in the relevant papers were also evaluated, and further articles were added if necessary.

We summarized the articles included in our literature review in Table 3.

**Table 3:** Articles included in the literature review

Number	Authors, year, country	Study type	Study period	*N*° of partecipants	Diagnosis and comorbidities	Prematurity	Intervention 1	Intervention 2	Intervention 3	Primary outcome	Secondary outcome	Results
11	Khalaf, 2001, USA	pRCT	Sep 1998- Sep 1999	64	RDS	< 34 wks GA	sNIPPV	nCPAP		extubation failure	apnea/bradycardia, PFT	sNIPPV prevent extubation failure, apnea and bradycardia
12	Moretti, 1999, Italy	rCOT	NA	11	RDS required MV, preterm < 1500 g	mean GA 28 wks	sNIPPV	nCPAP		ventilation, gas exchange and respiratory effort		sNIPPV provide more ventilatory support in the post-extubation period with less inspiratory effort
13	Barrington, 2001, USA	pRCT	Mar 1996 - Jan 1999	54	BW < 1251-g birth weight who extubated before 6 weeks of age	< 32 wks GA	sNIPPV	nCPAP		extubation failure		sNIPPV is effective in preventing extubation
14	Kulkarni, 2006, USA	rCCS	May 2000 - Dec 2003	60	RDS, requirement of at least 1 dose of surfactant	< 32 wks GA	sNIPPV	nCPAP		BPD, growth	NEC, ROP, LOS	SNIPPV resulted in decreased BPD, without affecting weight gain or the incidence of other short-term morbidities
15	Moretti, 2008, Italy	pRCT	Mar 2002 - Jun 2005	63	BW < 1251 g with RDS	< 32 wks GA	sNIPPV	nCPAP		extubation failure	days of invasive MV, days on oxygen, incidence of CLD, duration of hospital stay	sNIPPV prevent extubation failure
16	Aghai, 2006, USA	rCOT	Jan 2003 - Nov 2004	15	Infants < 2,000 g birth weight, requiring NCPAP for mild RDS.	<32 wks GA	sNIPPV	nCPAP		impact on WOB		sNIPPV decreases WOB in premature neonates with mild RDS
17	Gizzi, 2012, Italy	rCCS	Jan 2009 -Dec 2010	64	RDS required Surfactant	< 32 wks GA	nCPAP	sNIPPV		INSURE failure with need for reintubation	PDA, BPD, PNX, IVH, ROP, NEC, death	sNIPPV after INSURE reduced MV need and favorably affected short-term morbidities
18	Ding, 2020, China	pCS	Oct 2017–2018	120	RDS, required MV	<32 wks GA	sNIPPV	nCPAP	sNIPPV-nCPAP	extubation failure, success rate of weaning from noninvasive ventilation within one week, and time of noninvasive ventilation	Time of oxygen therapy, time to total enteral feeding, hospital stay and medical cost	sNIPPV group and the sNIPPV + nCPAP group had significantly higher rate of successful extubation and removal from non-invasive ventilation within 1 week
19	Bhandari, 2009, USA	rCCS	Jan 2002 - Dec 2004	469	Preterms with BW < 1250 g	mean GA 27 wks	sNIPPV	nCPAP		rates of BPD and BPD/death	NDI or NDI/death	sNIPPV was associated with decreased BPD, BPD/death, NDI, and NDI/death
20	Zheng, 2021, China	rCCS	Jan 2020 - Sep 2020	63	CHD	NO	sNIPPV	nCPAP		extubation failure	duration of postoperative NIV support, LOS, VAP, mortality, PNX, diaphragm and vocal cord, time to full gastrointestinal feeding, total cost	sNIPPV avoid reintubation after congenital heart surgery and significantly improved oxygenation and reduced PaCO2 retention after extubation
21	Chang, 2011, Taiwan	rCOT	/	16	clinically stable preterm infants	< 32 wks GA	nCPAP	sNIPPV	NIMV	Ventilation, gas exchange and WOB		No difference in ventilation and gas exchange. sNIPPV reduce WOB
22	Gizzi, 2015, Italy	rCOT	Oct 2010 - Feb 2012	19	AOP requiring NIV and supplemental FiO2	< 34 wks GA	sNIPPV	NIPPV	nCPAP	event rate of desaturations and bradycardias	central apnoeas, HR, FiO2, SpO2, transcutaneous blood gases and RR	Flow-sNIPPV seems more effective in reducing desaturations, bradycardias and central apnoea episodes
23	Salvo, 2015, Italy	pRCT	Jan 2010 - Dec 2012	124	Preterms VLBWI with RDS	< 32 wks GA	sNIPPV	BiPAP		Duration and failure of NIV support	Incidence of PNX, BPD, IVH, PVL, need and N° of surfactant doses, need for postnatal glucocorticoid treatment, PDA, ROP NEC, LOS, death, and days to regain BW	sNIPPV and BiPAP used as primary respiratory support in the treatment of RDS of VLBW infants are feasible and equally effective
24	Salvo, 2017, Italy	rCCS	Jan 2013 - Dec 2015	191	RDS, preterm < 1500 g	< 32 wks GA	nCPAP	sNIPPV	BiPAP	NIV failure in the first 5 days of life	surfactant treatment and doses, PDA, BPD, PNX, IVH, ROP, NEC, LOS, death	sNIPPV/ BiPAP augments the beneficial effects of nCPAP
25	Phatigomet, Thailand, 2024	pRCT	Jul 2020 - Jun 2022	133	All inborn preterm and term neonates requiring NIV after extubation	YES/NO	sNIPPV	nHFOV		reintubation rate within 7 days of nHFOV and sNIPPV	incidence of BPD, ROP, IVH, PVL, NEC stage II and III, duration of O2 use after extubation, hospital stay, and daily hospital cost	No difference between nHFOV and sNIPPV modes in reintubation rate
26	Atanasov, 2023, Germany	rCOT	NA	22	VLBW in NIV	< 32 wks GA	sNIPPV	nHFOV		time spent within the SpO2 target (SpO2 88–95%)	hypoxemia and in hyperoxemia; N° of bradycardia episodes, cerebral tissue oxygen saturation, RR, HR, transcutaneous pCO2 and FiO2	sNIPPV is more efficient than nHFOV to retain the SpO2 target and to reduce FiO2 exposure.
27	Baingam, 2024, Thailand	pRCT	Jul 2020 - Jun 2022	103	intubated neonates who needed NIV after extubation	YES/NO	sNIPPV	nHFOV		arterial pCO2 levels		pCO2 levels after 2 h of nHFOV were significantly higher than those after sNIPPV
28	Dumpa, 2012, USA	rCCS	Jan 2004- Dex 2009	410	Preterm and term neonates who received SNIPPV/NIPPV	YES/NO	sNIPPV	NIPPV		BPD/death	PDA, sepsis, IVH, PVL, ROP, NEC, days on ETT, days on NIV, days on nCPAP, days on TPN, lenght of stay	sNIPPV relative to NIPPV did not show a significantly different impact on clinical outcomes in premature infants.
29	Huang, 2015, Germany	rCOT	Jul 2012. Jun 2013	14	VLBW recovering from RDS, after extubation	< 32 wks GA /	sNIPPV	NIPPV		WOB, cerebral StO2, apnea		sNIPPV after extubation in VLBWI recovering from RDS improved gas exchange and decreased WOB
30	Ricotti, 2013, Italy	rCOS	Dec 2007 - Dec 2010	78	ELBW/VLBW with RDS	<32 wks GA	sNIPPV	BiPAP		duration and failure of NIV	incidence of PNX, CLD, BPD IVH, PVL, need and N° of dose of surfactant, need of postnatal glucocorticoids, PDA, ROP, NEC, LOS, death, weight gain, duration of MV, lenght of staying in hospital	No differences between sNIPPV and BiPAP in the treatment of RDS and in secondary outcomes
31	Charles, 2018, Germany	rCOT	NA	9	Neonates < 32 GA who had been mechanically ventilated for at least 48 h and requiring NIV post-extubation	< 32 wks GA	sNIPPV	HFT		WOB and TAA		sNIPPV compared to HFT significantly reduced the WOB and TAA in preterm infants immediately post-extubation
32	Bhandari, 2007, USA	pRCT	July 2000-March 2005	31	Preterm with BW of 600 to 1250 g post administration of Surfactant for RDS	mean GA 27 wks	sNIPPV	CV		BPD, death	PDA, IVH, PVL, NEC, ROP, NDI	sNIPPV reduces significantly incidence of BPD/death
43	Kiciman,1998, USA	rCCS	NA	14	RDS	< 36 wkg GA	ETT-CPAP	nCPAP	sNIPPV	TAM		sNIPPV reduces TAM compared to nasal-CPAP or ETT-CPAP

*pRCT* prospective randomized control trial, *rCOT* randomized cross-over trial, *rCCS* retrospective case-control study, *pCS* prospective cohort study, *TAA* thoracoabdominal asynchrony, *WOB* work of breathing, *BW* birth weight, *MV* mechanical ventilation, *VAP* ventilator-associated pneumonia, *RR* respiratory rate, *HR* heart rate, *AOP* apnoea of prematurity, *PDA* patent ductus arteriosus, *BPD* bronchopulmonary dysplasia, *CLD* chronic lung disease, *PNX* pneumothorax, *IVH* intraventricular hemorrhage, *ROP* retinopathy of prematurity, *NEC* necrotizing enterocolitis, *LOS* late onset sepsis, *PVL* periventricular leukomalacia, *TPN* total parenteral nutrition, *NDI* neurodevelopmental impairment, *ETT* endotracheal tube, *CV* conventional ventilation, *nCPAP* Nasal Continuous Positive Airway Pressure, *sNIPPV* synchronized nasal intermittent positive pressure ventilation, *NIPPV* nasal intermittent positive pressure ventilation, *BiPAP* bilevel positive Airway pressure, *nHFOV* nasal High Frequency Oscillatory Ventilation, *HFT* high flow therapy, *TAM* thoracoabdominal motion, *ETT* endotracheal tube, *wks* weeks, *GA* gestational age

Nine studies compared sNIPPV to the nCPAP in preterm infants [8–18]. All the authors supported the superiority of sNIPPV as a first-line ventilatory strategy and a noninvasive ventilation modality to avoid extubation failure.

Zheng et al. in 2021 compared sNIPPV and nCPAP to avoid extubation failure in neonates with congenital heart diseases in the post-operative period. Synchronized NIPPV proved superior to NCPAP in avoiding reintubation after congenital heart surgery and significantly improved oxygenation and reduced PaCO2 retention after extubation [19].

In 2011, Chang et al. conducted a randomized crossover trial in clinically stable preterm infants. They compared nCPAP, sNIPPV, and nasal Intermittent Mandatory Ventilation (NIMV), finding that ventilation and gas exchange did not improve during NIMV or SIPPV compared with nCPAP, but the synchronization reduced spontaneous breathing effort [20].

Gizzi et al., in 2014, with a similar study design, found that sNIPPV seems more effective than NIPPV and NCPAP in reducing the incidence of desaturations, bradycardias, and apnea of prematurity (AOP) [21].

Salvo et al. initially compared, in 2015 [22], sNIPPV and Bilevel Positive Airway Pressure (BiPAP), and subsequently, in 2017, sNIPPV, BiPAP, and nCPAP. In these trials, sNIPPV and BiPAP were equally superior to nCPAP in treating RDS of very low birth weight (VLBW) infants [23].

Three studies used nHFOV and the sNIPPV to prevent desaturation, extubation failure, and hypercapnia in preterm infants [24–26].

They found no difference in the overall reintubation rate between the nHFOV and sNIPPV modes [24]. However, sNIPPV appeared to be more effective in preventing episodes of desaturation [25] and hypercapnia [26]. Two studies, instead, compared NIPPV and sNIPPV in term, preterm, and, in particular, VLBW infants [27, 28]. 

Dumpa et al. found no significant differences in terms of BPD or death in the use of sNIPPV vs. NIPPV [27]. Huang et al. found that synchronization during nasal ventilation, immediately after extubation in VLBW infants recovering from RDS, improves gas exchange and decreases respiratory effort [28].

In only one study, sNIPPV was compared to BiPAP for the treatment of preterm neonates with RDS, showing no significant difference between the two ventilatory strategies [29]. Another study compared nasal High Flow Therapy (nHFT) with sNIPPV in infants with GA of less than 32 weeks [30] demonstrating that sNIPPV significantly reduced both the work of breathing (WOB) and thoracoabdominal asynchrony (TAA) immediately after extubation.

Early extubation to sNIPPV following surfactant administration in preterm infants seems moreover associated with a significantly lower incidence of BPD and mortality [31].

## Discussion

Newborns with CDH are extremely fragile due to the characteristics of the disease itself, and they are at high risk of VILI [5]. Therefore, weaning these patients from invasive ventilation as early as possible is crucial. However, they also present a high rate of extubation failure due to pre-existing lung hypoplasia and impaired diaphragm activity and motility.

Schroeder et al. reported an extubation failure rate of 35% [32]. Their study on clinical and echocardiographic predictors of reintubation risk in neonates with CDH did not identify the best ventilatory strategy to counteract the factors that make these infants more likely to experience extubation failure.

Our center also has no standard extubation protocol for these patients: the extubation is attempted when the baby shows an effective spontaneous respiratory drive, when blood gas analysis confirms adequate gas exchange, when LUS and chest x-ray rule out significant atelectasis or effusion, and when an echocardiogram performed in the previous 24–48 h excludes PH. Before extubation, we administer a dose of betamethasone to reduce laryngeal edema and a dose of caffeine to further stimulate respiratory drive.

The choice of NIV support is based on the clinical and instrumental characteristics of the individual patient, usually favoring nCPAP or NIPPV. A role in the selection of NIV support is also determined by the type of devices available in our hospital and by the knowledge and confidence of the staff.

To the best of our knowledge, this is the first description of the use of flow-sNIPPV as a post-extubation ventilatory strategy in neonates with CDH to date. While there is a great amount of literature regarding invasive mechanical ventilation strategies in patients with CDH at birth and during the pre-operative period, no guidelines tell us how to best ventilate these patients after extubation.

The CDH EURO Consortium indicates a standardized postnatal management approach recommending conventional mechanical ventilation as the optimal initial ventilation strategy and HFOV as a rescue therapy [33].

The literature consistently shows that permissive hypercapnia and ‘gentle ventilation’ increase survival in neonates with CDH [34, 35].

Sakai et al. indicate that after surgical repair, respiratory compliance decreases. This would justify the need to reduce PEEP levels to avoid alveolar overdistension. However, they are referring to invasive mechanical ventilation [36].

Patient-ventilator synchrony is fundamental in reducing major complications caused by asynchrony between the patient’s breathing effort and the ventilator’s mandatory breath [37].

Asynchronous cycles occurring late during spontaneous inspiration or expiration activate pulmonary stretch receptors, disrupting the infant’s natural breathing rhythm. The Hering-Breuer inflation reflex triggers provoke active expiratory efforts against the ventilator cycle, delaying spontaneous exhalation and the initiation of subsequent inspiration [37]. Additionally, asynchronous breaths may increase the activity of the glottal constrictor muscle due to stimulation of upper airway pressure receptors. This response can redirect airflow into the digestive tract [37]. Lastly, it is widely recognized that asynchrony during NIPPV can cause stress and distress, potentially compromising treatment success [38]. In our patients, sNIPPV was crucial in reducing WOB and in improving ventilation. With synchronization, positive pressure is efficiently transmitted to the lungs because it is delivered when the glottis is open, thus reducing the risk of abdominal overdistension [37], representing a goal in the post-operative period and, perhaps, even more after a CDH repair.

Synchronization increases transpulmonary pressure produced by the combination of the patient’s negative pressure and the ventilator’s positive pressure [39]. This leads to a stabilization of the chest wall, reducing TAA [40] and WOB.

This effect could be even more helpful in neonates with CDH, who often have coexisting chest wall abnormalities and weakness of accessory respiratory muscles.

In our center, we performed sNIPPV with the Giulia^®^ ventilator, which allows synchronization via a high-precision, low-resistance flow sensor placed proximal to the Y-piece. This advanced flow sensor is designed and developed to overcome the problems of instability and reliability of the flow signal caused mainly by continuous and variable leaks from the open circuit. Bench tests have demonstrated that this device can detect very small inspiratory flow and volumes, that its performance is not affected by the size of leaks, and that its response time is < 100 ms [37].

To promote patient comfort, a double inspiratory loop cannula characterized by low resistance (Sync-flow-cannula ^®^) was used as an interface [41], and the initial ventilatory parameters were set according to current literature recommendations [37].

In all our cases, sNIPPV proved more effective than nIPPV in avoiding reintubation by improving respiratory dynamics, lung ventilation, and gas exchange. Moreover, the synchronization permitted a much more rapid and easier reduction of the analgosedation thanks to the gain in comfort that this ventilatory mode entails.

Although there is no literature on the use of sNIPPV in neonates with CDH to confirm our data, this ventilatory strategy has been widely studied in other populations of neonates, particularly in preterm infants.

As early as 1998, Kiciman et al. demonstrated that nasal synchronized intermittent mandatory ventilation reduced asynchronous thoracoabdominal motion compared to nCPAP, thanks to the chest wall stabilization [42].

Subsequently, in 1999, Moretti et al. compared the effects of sNIPPV versus nCPAP in ventilation, gas exchange, and patient inspiratory effort in 11 preterm neonates immediately after extubation. The results showed that sNIPPV improves pulmonary function and decreases respiratory effort after extubation in VLBW infants [11].

These results were confirmed by Aghai in 2006 and Chang in 2011, with particular attention to the reduction of WOB that synchronization entailed [15, 20].

SNIPPV has also been used for purposes other than post-extubation respiratory support.

Gizzi et al. in 2015, in a population of 19 newborns with an average GA of 30 weeks, described how sNIPPV was more effective than nCPAP and NIPPV in reducing episodes of AOP, desaturation, and bradycardia. They believed that flow-sNIPPV limited oxygenation instability by reducing the incidence of AOP or stabilizing the functional residual capacity (FRC), which preterm babies easily lose [21].

In our patients, we did not observe an improvement in saturation values since they remained stable even during NIPPV. However, the improved lung aeration observed through LUS certainly reflects an increase in FRC.

We can speculate that the weakness of the accessory respiratory muscles in our patients, as well as the poor compliance of the hypoplastic lung, may benefit from synchronization due to the same physiological mechanisms seen in preterm infants.

The limitations of our study include the small sample size and the retrospective design, without a specific weaning protocol. However, we believe that our study’s limitations can be partially counterbalanced by the technical and pathophysiological premises underlying synchronized ventilation and how this can positively benefit the relationship between the neonate with CDH and the ventilator. It should be underlined that in all our cases, sNIPPV was introduced only after extubation failure with NIPPV, and thus it was never used as a first-line strategy. This represents a potential bias, as the apparent benefits we observed may also be partially related to the timing of its introduction. For this reason, future prospective and comparative studies are needed to better clarify the effectiveness of sNIPPV as both early and a rescue modality in neonates with CDH.

Moreover, pleural effusion was present in three of the four cases (P1, P3, and P4), while P2 did not develop significant effusion. In patients without substantial effusion, such as P2, NAVA could be an effective alternative. We hope that future prospective studies will directly compare sNIPPV and NAVA to determine the optimal ventilatory strategy in neonates with CDH, particularly in the absence of pleural effusion.

## Conclusion

To the best of our knowledge, this is the first report on the effectiveness of flow-sNIPPV in reducing extubation failure in neonates with surgically corrected CDH. Although not conclusive, our findings seemed to indicate that flow-sNIPPV could improve extubation success in CDH infants, reducing thoraco-abdominal asynchrony and WOB and thus improving lung ventilation and gas exchange. At the same time, this non-invasive mode of respiratory care does not increase the risk of ventilation-related complications.

This report can be a starting point for building new prospective studies and confirming these findings.

## Data Availability

Not applicable.

## Abbreviations

AOP: Apnea of Prematurity
BiPAP: Bilevel Positive Airway Pressure
BPD: Bronchopulmonary Dysplasia
BUR: Back-Up Rate
CDH: Congenital Diaphragmatic Hernia
ECMO: Extracorporeal Membrane Oxygenation
FRC: Functional Residual Capacity
GA: Gestational Age
HFOV: High-Frequency Oscillatory Ventilation
IT: Inspiratory Time
LHR O/E: Observed-to-Expected Lung-to-Head Ratio
LUS: Lung Ultrasonography
MAP: Mean Airway Pressure
MRI: Magnetic Resonance Imaging
MSA: Mediastinal Shift Angle
NAVA: Neurally Adjusted Ventilatory Assist
NIV: Non-Invasive Ventilation
NIPPV: Nasal Intermittent Positive Pressure Ventilation
nCPAP: Nasal Continuous Positive Airway Pressure
nHFOV: Nasal High-Frequency Oscillatory Ventilation
nHFT: Nasal High Flow Therapy
PH: Pulmonary Hypertension
PEEP: Positive End-Expiratory Pressure
PIP: Peak Inspiratory Pressure
RR: Respiratory Rate
RDS: Respiratory Distress Syndrome
sNIPPV: Synchronized Nasal Intermittent Positive Pressure Ventilation
SIPPV: Synchronized Intermittent Positive Pressure Ventilation
TAA: Thoraco-Abdominal Asynchrony
VA-ECMO: Veno-Arterial Extracorporeal Membrane Oxygenation
VILI: Ventilator-Induced Lung Injury
VLBW: Very Low Birth Weight
WOB: Work of Breathing
